# Supplementary material for: Risk factors for aggravated COVID-19 despite medical care after admission among Japanese patients: A Japanese association for infectious diseases COVID registry study
Source: PLoS One. 2025 Oct 30;20(10):e0335439. doi: 10.1371/journal.pone.0335439 (PMC12574867; doi:10.1371/journal.pone.0335439)
Supplement: S1 Table — (DOCX) [file pone.0335439.s001.docx]

S1 Table. Univariate analysis of aggravating factors related to patient demographics (FAS 2)

| Variable |  | N | Unadjusted odds ratio | 95% confidence interval | P-value |
| --- | --- | --- | --- | --- | --- |
| Age (years) | <65 (ref.) | 1,525 |  |  |  |
|  | 65–<75 | 429 | 6.13* | 3.95–9.52 | <0.001 |
|  | ≥75 | 644 | 6.12* | 4.07–9.21 | <0.001 |
| Sex | Male (ref.) | 1,520 |  |  |  |
|  | Female | 1,078 | 0.68* | 0.49–0.94 | 0.020 |
| BMI (kg/m^2^) | <18.5 | 247 | 0.61 | 0.32–1.17 | 0.139 |
|  | 18.5–24.9 (ref.) | 1,338 |  |  |  |
|  | ≥25 | 770 | 1.01 | 0.71–1.43 | 0.965 |
| Pregnancy | Yes/No (ref.) | 27/1,042 | 0.69 | 0.09–5.18 | 0.719 |
| Smoking | Yes/No (ref.) | 906/1,184 | 1.92* | 1.33–2.78 | <0.001 |
| Time of admission | Stage I (ref.) | 483 |  |  |  |
|  | Stage II | 716 | 0.36* | 0.22–0.60 | <0.001 |
|  | Stage III | 1,399 | 0.78 | 0.54–1.12 | 0.121 |
| Diabetes | Yes/No (ref.) | 533/1,939 | 3.07* | 2.21–4.26 | <0.001 |
| Hypertension | Yes/No (ref.) | 886/1,702 | 3.35* | 2.43–4.62 | <0.001 |
| Hyperlipidemia | Yes/No (ref.) | 486/1,847 | 1.88* | 1.33–2.65 | <0.001 |
| Allergic diseases | Yes/No (ref.) | 163/2,100 | 0.48 | 0.21–1.09 | 0.080 |
| Heart valve diseases | Yes/No (ref.) | 41/2,197 | 2.71* | 1.18–6.22 | 0.018 |
| Congestive heart disease | Yes/No (ref.) | 88/2,161 | 1.89 | 0.98–3.63 | 0.056 |
| Peripheral arterial diseases | Yes/No (ref.) | 27/2,217 | 6.70 | 2.96–15.16 | <0.001 |
| Atrial fibrillation | Yes/No (ref.) | 110/2,142 | 1.30 | 0.67–2.54 | 0.443 |
| Aortic diseases | Yes/No (ref.) | 22/2,223 | 2.05 | 0.60–7.00 | 0.252 |
| Chronic obstructive pulmonary disease | Yes/No (ref.) | 91/2,480 | 3.80* | 2.21–6.54 | <0.001 |
| Interstitial pneumonia | Yes/No (ref.) | 31/2,441 | 3.14* | 1.27–7.77 | 0.013 |
| Asthma | Yes/No (ref.) | 185/2,386 | 1.08 | 0.60–1.94 | 0.796 |
| Pulmonary aspergillosis | Yes/No (ref.) | 6/2,263 | 26.11* | 4.75–143.64 | <0.001 |
| Renal failure | Yes/No (ref.) | 151/2,129 | 4.04* | 2.63–6.19 | <0.001 |
| Hemodialysis | Yes/No (ref.) | 85/2,129 | 4.38* | 2.58–7.43 | <0.001 |
| Immunodeficiency syndrome | Yes/No (ref.) | 50/2,320 | 1.16 | 0.41–3.26 | 0.780 |
| Collagen diseases | Yes/No (ref.) | 64/2,226 | 2.46* | 1.23–4.92 | 0.011 |
| Malignancy, cured | Yes/No (ref.) | 134/2,450 | 1.16 | 0.60–2.26 | 0.658 |
| Malignancy, active | Yes/No (ref.) | 111/2,478 | 2.77* | 1.61–4.76 | <0.001 |
| Prostate cancer, active | Yes/No (ref.) | 12/2,579 | 7.32* | 2.18–24.55 | 0.001 |
| Bladder cancer, active | Yes/No (ref.) | 6/2,585 | 14.57* | 2.92–72.76 | 0.001 |
| Multiple myeloma, active | Yes/No (ref.) | 6/2,585 | 7.25* | 1.32–39.84 | 0.023 |
|  |  |  |  |  |  |
| Calcineurin inhibitors | Yes/No (ref.) | 20/2,506 | 0.00 | 0–∞ | 0.982 |
| Corticosteroids | Yes/No (ref.) | 124/2,403 | 3.57* | 2.20–5.79 | <0.001 |
| Hypertension drugs | Yes/No (ref.) | 807/1,764 | 2.94* | 2.14–4.06 | <0.001 |
| Hyperlipidemia drugs | Yes/No (ref.) | 435/2,145 | 1.85* | 1.29–2.65 | <0.001 |
| Chemotherapy | Yes/No (ref.) | 41/2,538 | 3.06* | 1.34–7.01 | 0.008 |
| Diabetes drugs | Yes/No (ref.) | 335/2,243 | 2.75* | 1.91–3.95 | <0.001 |
| Anticoagulants | Yes/No (ref.) | 133/2,458 | 1.45 | 0.79–2.69 | 0.233 |
| Antiplatelet drugs | Yes/No (ref.) | 221/2,372 | 3.01* | 2.01–4.52 | <0.001 |
| Estrogen agonists | Yes/No (ref.) | 23/2,562 | 0.00 | 0– | 0.981 |
| Immune checkpoint inhibitors | Yes/No (ref.) | 6/2,586 | 0.00 | 0– | 0.985 |
| Antirheumatic drugs | Yes/No (ref.) | 33/2,493 | 3.22* | 1.31–7.91 | 0.011 |
| Allergy drugs | Yes/No (ref.) | 106/2,477 | 1.35 | 0.67–2.73 | 0.398 |

*Statistically significant (p<0.05), BMI; body mass index, GCS; Glasgow Coma Scale
