# Supplementary material for: Risk factors for aggravated COVID-19 despite medical care after admission among Japanese patients: A Japanese association for infectious diseases COVID registry study
Source: PLoS One. 2025 Oct 30;20(10):e0335439. doi: 10.1371/journal.pone.0335439 (PMC12574867; doi:10.1371/journal.pone.0335439)
Supplement: S2 Table — (DOCX) [file pone.0335439.s002.docx]

S2 Table. Univariate analysis of symptom onset and physical findings on admission (FAS 2)

| Variable |  | N | Unadjusted odds ratio | 95% confidence interval | P-value |
| --- | --- | --- | --- | --- | --- |
| Symptoms at disease onset |  |  |  |  |  |
| Fever | Yes/No (ref.) | 1,832/360 | 1.81* | 1.06–3.07 | 0.029 |
| Cough | Yes/No (ref.) | 915/1,250 | 1.21 | 0.87–1.67 | 0.257 |
| Breathing difficulty | Yes/No (ref.) | 258/1,861 | 3.21* | 2.20–4.68 | <0.001 |
| Fatigue | Yes/No (ref.) | 673/1,475 | 1.13 | 0.80–1.59 | 0.496 |
| Olfactory disturbance | Yes/No (ref.) | 138/1,983 | 0.18* | 0.04–0.87 | 0.015 |
| Taste disturbance | Yes/No (ref.) | 175/1,943 | 0.35* | 0.14–0.87 | 0.024 |
| Consciousness disturbance | Yes/No (ref.) | 20/2,089 | 1.41 | 0.32–6.12 | 0.648 |
| Diarrhea | Yes/No (ref.) | 136/1,982 | 0.78 | 0.70–1.63 | 0.508 |
| Physical findings on admission |  |  |  |  |  |
| Body temperature |  | 2,595 | 1.03* | 1.02–1.03 | <0.001 |
| SpO_2_ |  | 2,590 | 0.97* | 0.96–0.98 | <0.001 |
| Consciousness category, JCS | 0 (ref.) | 2,070 |  |  |  |
|  | Category I | 128 | 3.51* | 2.16–5.70 | <0.001 |
|  | Category II | 26 | 6.07* | 2.52–14.58 | <0.001 |
|  | Category III | 8 | 15.77* | 3.90–63.82 | <0.001 |
| Consciousness score, GCS |  | 2,141 | 0.98* | 0.98–0.99 | <0.001 |

*Statistically significant (p<0.05), JCS: Japan Coma Scale, Category I: delirium/confusion/senselessness, Category II: stupor/lethargy/hypersomnia/somnolence/drowsiness, Category III: deep coma/coma/semi-coma; GCS: Glasgow Coma Scale
