# Supplementary material for: Risk factors for aggravated COVID-19 despite medical care after admission among Japanese patients: A Japanese association for infectious diseases COVID registry study
Source: PLoS One. 2025 Oct 30;20(10):e0335439. doi: 10.1371/journal.pone.0335439 (PMC12574867; doi:10.1371/journal.pone.0335439)
Supplement: S3 Table — (DOCX) [file pone.0335439.s003.docx]

S3 Table. Univariate analysis of laboratory findings on admission (FAS 2)

| Variable | | N | Unadjusted odds ratio | 95% confidence interval | P-value |
| --- | --- | --- | --- | --- | --- |
| White blood cells | | 2,548 | 1.03* | 1.02–1.04 | <0.001 |
| Neutrophils | | 2,474 | 1.06* | 1.05–1.07 | <0.001 |
| Eosinophils | | 2,441 | 0.74* | 0.68––0.80 | <0.001 |
| Monocytes | | 2,133 | 0.97* | 0.96–0.98 | <0.001 |
| Lymphocytes | | 2,481 | 0.94* | 0.93–0.95 | <0.001 |
| Red blood cells | | 2,224 | 0.97* | 0.96–0.98 | <0.001 |
| Hemoglobin | | 2,546 | 0.98* | 0.97–0.98 | <0.001 |
| Hematocrit | | 2,228 | 0.98* | 0.97–0.99 | <0.001 |
| Platelets | | 2,548 | 1.00 | 0.99–1.00 | 0.427 |
| Total protein | | 2,049 | 0.97* | 0.96–0.97 | <0.001 |
| Albumin | | 2,469 | 0.95* | 0.95–0.96 | <0.001 |
| Aspartate aminotransferase | | 2,548 | 1.00 | 1.00–1.01 | 0.139 |
| Alanine aminotransferase | | 2,543 | 1.00 | 1.00–1.01 | 0.426 |
| Alkaline phosphatase | | 2,177 | 1.00 | 0.99–1.01 | 0.559 |
| γ–glutamyl transpeptidase | | 2,391 | 1.01* | 1.00–1.01 | 0.024 |
| Lactate dehydrogenase | | 2,530 | 1.04 * | 1.02–1.05 | <0.001 |
| Total bilirubin | | 2,475 | 1.00 | 0.99–1.01 | 0.728 |
| Blood urea nitrogen | | 2,540 | 1.03* | 1.02–1.04 | <0.001 |
| Creatinine | | 2,541 | 1.02* | 1.01–1.02 | <0.001 |
| Estimated glomerular filtration rate | | 1,915 | 0.98* | 0.97–0.99 | <0.001 |
| C-reactive protein | | 2,426 | 1.10* | 1.08–1.12 | <0.001 |
| Prothrombin time international normalized ratio | | 2,026 | 1.00 | 0.99–1.01 | 0.341 |
| Activated partial thromboplastin time | | 1,902 | 1.01* | 1.00–1.02 | 0.009 |
| Fibrinogen | | 1,428 | 1.01* | 1.00–1.02 | 0.008 |
| D-dimer | | 2,178 | 1.00 | 0.99–1.01 | 0.868 |
| Procalcitonin | | 994 | 1.00 | 0.99–1.01 | 0.961 |
| Ferritin | | 1,282 | 1.02* | 1.01–1.03 | <0.001 |
|  | |  |  |  |  |
| HbA1c | <6.5 (ref.) | 1,025 |  |  |  |
|  | 6.5–8.0 | 290 | 2.45 | 1.58–3.81 | <0.001 |
|  | ≥8.0 | 152 | 3.09* | 1.84–5.18 | <0.001 |

*Statistically significant (p<0.05)
