# Supplementary material for: Risk factors for aggravated COVID-19 despite medical care after admission among Japanese patients: A Japanese association for infectious diseases COVID registry study
Source: PLoS One. 2025 Oct 30;20(10):e0335439. doi: 10.1371/journal.pone.0335439 (PMC12574867; doi:10.1371/journal.pone.0335439)
Supplement: S4 Table — (DOCX) [file pone.0335439.s004.docx]

S4 Table. List if 36 institutions throughout Japan that joined the JAID-COVID registry.

| Institution/Hospital |  |
| --- | --- |
| Saitama Medical University | Fujita Health University |
| National Hospital Organization Tokyo Medical Center | Sanuki Municipal Hospital |
| Tokyo Medical University Hospital | Tome Citizen Hospital |
| Jikei University | Saiseikai Kumamoto Hospital |
| Tokyo Metropolitan Bokutoh Hospital | JCHO　Hokkaido Hospital |
| Toranomon Hospital | Nagasaki University Hospital |
| Yokohama City Minato Red Cross Hospital | Shimonoseki City Hospital |
| Tosei General Hospital | JCHO Tokyo Takanawa Hospital |
| Japanese Red Cross Nagoya Daini Hospital | Oita Prefectural Hospital |
| Wakayama Medical University Hospital | Yamagata University Hospital |
| National Hospital Organization Kyoto Medical Center | Japanese Red Cross Asahikawa Hospital |
| Kyoto City Hospital | Osaka Medical and Pharmaceutical University Hospital |
| Osaka City General Hospital | NHO Kumamoto Saishun Medical Center |
| Nara Medical University | Keio University School of Medicine |
| Tohoku Medical and Pharmaceutical University Hospital. | Saitama Medical University International Medical Center |
| Tohoku University Hospital | Yokohama City University Hospital |
| University of Ryukyu Hospital | Shimane University Hospital |
| Self-Defense force Central Hospital | Osaka Saiseikai Nakatsu Hospital |
