# Supplementary material for: Risk factors for aggravated COVID-19 despite medical care after admission among Japanese patients: A Japanese association for infectious diseases COVID registry study
Source: PLoS One. 2025 Oct 30;20(10):e0335439. doi: 10.1371/journal.pone.0335439 (PMC12574867; doi:10.1371/journal.pone.0335439)
Supplement: Attachment — Submitted filename: Response to Reviewers.docx [file pone.0335439.s006.docx]

**PONE-D-24-06564**

**Risk factors for aggravated COVID-19 despite medical care after admission among Japanese patients: A Japanese Association for Infectious Diseases COVID registry study**

**PLOS ONE**

**There are many similar publications related with this topic.**

**I suggest some points for improvement.**

**- Please highlight the prognostic factors in your conclusion abstract. This study determined the aggravating factors for severe COVID-19 development despite medical care after admission in Japanese people during the Wuhan clone pandemic. Please be detailed in the aggravating factors.**

Ans.) Thank you for this suggestion. We have modified the abstract to clarify the aggravating factors.

“**Results**

A total of 2,884 patients were enrolled at 36 institutions throughout Japan. After excluding 25 ineligible patients and 45 protocol deviations, 2,814 patients were analyzed. In multivariate analysis to explore aggravating factors, the time of admission, age, body mass index (BMI), body temperature, hemoglobin, hematocrit, eosinophil, γGTP, LDH, and BUN were identified as factors.

**Conclusion**

This study determined the aggravating factors for severe COVID-19 development despite medical care after admission in Japanese people during the Wuhan clone pandemic. The aggravating factors were admission at the beginning of the pandemic, older age, high BMI, high body temperature, anemia, cholestasis, and dehydration.”

**- The method should be detail. Is this retrospective cohort?**

Ans.) Thank you for the suggestion. This was a retrospective cohort study. We have modified the abstract and the main text to clarify the study design.

Abstract

“**Materials and methods**

This was a retrospective, multicenter cohort study using the Japanese Association for Infectious Diseases COVID registry to explore aggravating factors for severe COVID-19 development despite medical care after admission in Japanese people. The study population comprised Japanese patients definitively diagnosed with COVID-19 between January 2020 and March 2021, the period of the Wuhan clone pandemic.”

Main body

“**Patients and study design**

This was a retrospective, multicenter cohort study of aggravating factors in Japanese COVID-19 patients. It used the COVID registry of the Japanese Association for Infectious Diseases (JAID), joined by 36 institutions throughout Japan.”

**- Please highlight the table.**

Ans.) Thank you for the suggestion. We have highlighted all columns with statistical significance using bold font.

**- Please begin the discussion with the main findings of your study.**

Ans.) Thank you for this suggestion. We have modified the abstract to clarify the aggravating factors.

“**Discussion**

This study determined the aggravating factors for severe COVID-19 development despite medical care after admission in Japanese people during the Wuhan clone pandemic. The aggravating factors were identified as admission at the beginning of the pandemic, older age, high BMI, high body temperature, anemia, cholestasis, and dehydration.”

**- Do the authors perform adjusted analysis? Please elaborate more.**

Ans.) Thank you for your comments regarding the adjusted analysis. We apologize for not making this clear in the manuscript. We performed adjusted analyses using two multivariate logistic regression models to examine the association between various variables and the risk of aggravation.

The first model (Model I) included objective indicators such as measurable data from laboratory findings or objective data that could be obtained even if a patient interview was not possible at admission. The second model (Model II) included information obtained from patient interviews at admission and from referring doctors when laboratory testing was not available.

For both models, we selected variables with a p-value of <0.1 and a missing rate of <30% based on the univariate analysis, as well as clinically important variables known to be associated with the outcome. We employed multiple imputation to handle missing data.

The adjusted odds ratios (ORs) and their 95% confidence intervals (CIs) were reported for each variable included in the models. We have modified the manuscript to provide a clearer explanation of the adjusted analysis in the Statistical analysis section.

**Statistical analysis**

“In addition, two multivariate logistic regression models for FAS 2 were used to examine the regression coefficient (OR) of each variable for aggravation. The first model (Model I) estimated the risk of aggravation based on objective indicators such as measurable data from laboratory findings or objective data, even if a patient interview could not be performed at admission. The second model (Model II) estimated the risk of aggravation based on patient interviews at admission and information from referring doctors in situations without laboratory testing. For both models, variables with a p-value of <0.1 and a missing rate of <30% based on the univariate analysis and known clinically important variables were included. The adjusted OR and 95% CI were reported. Multiple imputation was adopted for missing data. P-values of less than 0.05 were considered statistically significant.”

**- The conclusion should be concise and clear.**

Ans.) Thank you for this suggestion. We have modified the conclusions of the abstract and main text to clarify the aggravating factors.

Abstract

**“Conclusion**

This study determined the aggravating factors for severe COVID-19 development despite medical care after admission in Japanese people during the Wuhan clone pandemic. The aggravating factors were admission at the beginning of the pandemic, older age, high BMI, high body temperature, anemia, cholestasis, and dehydration.”

Body

“**Conclusion**

This study examined the aggravating factors for severe COVID-19 development despite medical care after admission in Japanese people during the Wuhan clone pandemic period. The aggravating factors were identified as admission at the beginning of the pandemic, older age, high BMI, high body temperature, anemia, cholestasis, and dehydration.”

**Journal Requirements:**

**When submitting your revision, we need you to address these additional requirements.**

**1. Please ensure that your manuscript meets PLOS ONE's style requirements, including those for file naming. The PLOS ONE style templates can be found at**

**https://journals.plos.org/plosone/s/file?id=wjVg/PLOSOne_formatting_sample_main_body.pdf and**

**https://journals.plos.org/plosone/s/file?id=ba62/PLOSOne_formatting_sample_title_authors_affiliations.pdf**

　　　Ans). We apologize for not following these journal requirements. We have made all the required modifications.

**2. Please provide additional details regarding participant consent. In the ethics statement in the Methods and online submission information, please ensure that you have specified what type you obtained (for instance, written or verbal, and if verbal, how it was documented and witnessed). If your study included minors, state whether you obtained consent from parents or guardians. If the need for consent was waived by the ethics committee, please include this information.**

Ans.) Thank you for your comments. Because the need for consent was waived by the ethics committee, we have added the following content.

Main text

“**Ethical considerations**

This study was approved by the Tohoku University Ethics Committee and the institutional review boards of the collaborating institutes (UMIN registration number 000043802). The need for participant consent was formally waived by the Ethics Committee because the cohort study was retrospective and exploratory.”

“**Ethics approval and consent to participate**

This study was approved by the Ethics Committee of Tohoku University (approval number T2020-0161). The need for participant consent was formally waived by the Ethics Committee. This study was performed following the Declaration of Helsinki of 1964 and its later amendments.”

**3. Please note that funding information should not appear in any section or other areas of your manuscript. We will only publish funding information present in the Funding Statement section of the online submission form. Please remove any funding-related text from the manuscript.**

Ans). We apologize for not meeting this journal requirement. We have deleted the funding information from the main text.

**4. Thank you for stating the following financial disclosure:**

**"This JAID-COVID registry study was conducted as a JAID joint research project with research funding from Astellas Pharma. "Please state what role the funders took in the study. If the funders had no role, please state: "The funders had no role in study design, data collection and analysis, decision to publish, or preparation of the manuscript." If this statement is not correct you must amend it as needed. Please include this amended Role of Funder statement in your cover letter; we will change the online submission form on your behalf.**

　　Ans.) Thank you for these comments. The funders had a role in the methodology and data validation. We have included this information in the cover letter.

**5. PLOS requires an ORCID iD for the corresponding author in Editorial Manager on papers submitted after December 6th, 2016. Please ensure that you have an ORCID iD and that it is validated in Editorial Manager. To do this, go to ‘Update my Information’ (in the upper left-hand corner of the main menu), and click on the Fetch/Validate link next to the ORCID field. This will take you to the ORCID site and allow you to create a new iD or authenticate a pre-existing iD in Editorial Manager. Please see the following video for instructions on linking an ORCID iD to your Editorial Manager account: https://www.youtube.com/watch?v=_xcclfuvtxQ**

　　Ans.) I have updated my ORCID iD information.

**6. One of the noted authors is a group or consortium "the COVID registry of the Japanese Association for Infectious Diseases". In addition to naming the author group, please list the individual authors and affiliations within this group in the acknowledgments section of your manuscript. Please also indicate clearly a lead author for this group along with a contact email address.**

Ans.) I have now added information on the Ad Hoc Committee managing the COVID registry of the Japanese Association for Infectious Diseases.

“**Acknowledgments**

Regarding data registration, we express our gratitude to the facility research managers of the 36 participating hospitals throughout Japan in the COVID registry of the JAID, listed in Table S4. The Ad Hoc Committee for the COVID-19–Aggravating Factors Search Project of the JAID managed this registry, led by Kazuyoshi Kawakami (Former professor of Tohoku University, Hirose Hospital, kawakami@med.tohoku.ac.jp). The other members are Itaru Nakamura (Tokyo Medical University Hospital), Yusuke Koizumi (Wakayama Medical University), Hideki Araoka (Toranomon Hospital), and Hiroaki Hata (National Hospital Organization Kyoto Medical Center).”

**7. Please include your tables as part of your main manuscript and remove the individual files. Please note that supplementary tables (should remain/ be uploaded) as separate "supporting information" files**

Ans.) We apologize for not following the journal requirement. We have now added the tables in the main text.

**8. Please include captions for your Supporting Information files at the end of your manuscript, and update any in-text citations to match accordingly. Please see our Supporting Information guidelines for more information: http://journals.plos.org/plosone/s/supporting-information.**

Ans.) We apologize for not meeting this journal requirement. We have deleted the supplemental table and added the supporting information in the main text.
