# Supplementary material for: Risk factors for aggravated COVID-19 despite medical care after admission among Japanese patients: A Japanese association for infectious diseases COVID registry study
Source: PLoS One. 2025 Oct 30;20(10):e0335439. doi: 10.1371/journal.pone.0335439 (PMC12574867; doi:10.1371/journal.pone.0335439)
Supplement: Attachment — Submitted filename: Response to reviewers_R2.docx [file pone.0335439.s007.docx]

**Response to reviewers**

**Reviewer #1:**

**In this manuscript, the authors aim to evaluate the aggravating factors contributing to severe COVID-19 progression despite medical care after hospital admission in Japanese individuals during the Wuhan strain pandemic, considering various risk factors. The study addresses an important issue regarding the use of laboratory parameters to assess disease worsening after admission. However, the manuscript presents a rather superficial analysis rather than serving as a robust reference. In particular, the study has critical flaws in its design, particularly in the methodology, and lacks important data. The authors should address the following points:**

→ We appreciate your detailed and constructive feedback. We have carefully considered each point and revised the manuscript accordingly. Below, we provide specific responses and indicate where corresponding revisions have been made in the manuscript.

1. **While the introduction provides an acceptable scientific background, it would be beneficial to better highlight the distinctiveness of the study and discuss other potential confounders not included in the analysis.**

→Thank you for this valuable suggestion. Previous studies have primarily identified risk factors in patients who were already in severe condition at the time of hospitalization or when they initially received medical care. In contrast, our study is distinctive in its focus on identifying risk factors for clinical deterioration despite the prevision of appropriate medical treatment. To better reflect this unique contribution, we have revised and streamlined the introduction, removed redundant background information on COVID-19, and emphasized our study’s novel focus.

Revised text (Lines 53–75):

**Introduction**

“Coronavirus disease 2019 (COVID-19) is caused by severe acute respiratory syndrome coronavirus 2 (SARS-CoV-2). The global mortality rate was particularly high at the onset of the pandemic, dominated by the Wuhan strain and subsequently by the Alpha and Delta variants. Mortality rates in the Japanese population have been reported to be among the lowest worldwide. However, during the Wuhan strain period—when vaccines were not yet available—some patient groups in Japan experienced disease worsening associated with common factors and comorbidities.

Previous studies from multiple countries have identified various factors influencing COVID-19 prognosis. Reported risk factors include age, sex, number and type of comorbidities, respiratory rate, body mass index (BMI), peripheral oxygen saturation, and level of consciousness, as measured by tools such as the Glasgow Coma Scale (GCS). Laboratory parameters reported as prognostic indicators include white blood cell (WBC) count, lymphocyte count, blood glucose, albumin (ALB), aspartate aminotransferase (AST), lactate dehydrogenase (LDH), creatinine kinase, urea, C-reactive protein (CRP), troponin I, procalcitonin, and D-dimer (1–10). However, these risk factors have generally been associated with patients already in severe condition upon hospital admission rather than with disease aggravation occurring despite medical care after admission. Moreover, most studies investigating risk factors for severe disease progression or death in COVID-19 patients have focused on non-Japanese populations. Data on COVID-19 outcomes specific to Japanese patients remain limited to a few reports (11–14).

In this study, we aimed to identify factors associated with COVID-19 aggravation during inpatient treatment despite receiving medical care, focusing on the Wuhan strain–dominant period in an unvaccinated Japanese population.”

2. **Another major concern is why the authors did not include age, sex, BMI, smoking status, and other risk factors alongside laboratory findings in the composite models, especially given the results of the univariate analysis. Additionally, why did the authors not use binary logistic regression with forward stepwise selection to assess independent predictors?**

→ Thank you for your important comment. We would like to clarify that age, sex, BMI, smoking status, and several laboratory variables were included in both the univariate and multivariate analyses. These results were provided in Supplementary Tables 1 and 3. However, we acknowledge that the supplemental placement may have limited their visibility. Based on the univariate analysis, variables with statistically significant associations—such as WBC, hemoglobin, albumin, LDH, and renal function—were included in the multivariate model. Although BMI did not reach statistical significance in the univariate analysis, we included it in the multivariate model due to its well-established clinical importance.

Regarding your suggestion to use binary logistic regression with forward stepwise selection, we did consider this approach during our initial analysis planning. However, our dataset contained missing values with varying degrees of completeness across variables. We were concerned that an automatic stepwise method might yield unstable or biased results depending on the variable inclusion sequence and missing data distribution. Therefore, we opted for a full model, selecting variables based on clinical relevance and *p*-values, to maintain model stability and interpretability.

Revised text (Lines 156–160):

“Although the forward stepwise method is commonly used for variable selection, the presence of variable missingness and varying missing rates raised concerns about model instability and bias from automated selection. Therefore, variables considered clinically important or with significant p-values were selected a priori, and a full-model approach including all these variables was adopted.”

3. **How did the authors evaluate the correlation between PLR or NLR and the laboratory findings, as well as other biomarkers such as WBC, HGB, ALB, and D-dimer? I suggest performing a Spearman’s correlation analysis including all factors and presenting a correlation matrix, such as a correlogram or heatmap, in the Results section.**

→ Thank you for your insightful suggestion. In response, we calculated the platelet-to-lymphocyte ratio (PLR) and neutrophil-to-lymphocyte ratio (NLR) for Model 1 of FAS2 cohort (Table 2 in the manuscript). We then created a correlogram using these and all clinical test-related variables. This explanation has also been added to the revised Methods and Results section.

Revised text (Lines 143–146):

**Statistical Analysis**

“Spearman’s correlation analysis was performed to evaluate associations between platelet-to-lymphocyte ratio (PLR) (16, 17), neutrophil-to-lymphocyte ratio (NLR) (16, 18), and laboratory findings, with correlograms constructed for all clinical test variables.”

Revised text (Lines 228–243):

**Results**

“The correlogram showing the correlations between the platelet-to-lymphocyte ratio (PLR), neutrophil-to-lymphocyte ratio (NLR), and other laboratory findings is presented in Figure 2.

**Figure 2.** Correlogram illustrating the correlations between laboratory findings and measurable clinical parameters.

**Figure legend:** GCS; Glasgow Coma Scale, WBC; white blood cells, RBC; red blood cells, HGB; hemoglobin, HCT; hematocrit, PLAT; platelets, AST; aspartate aminotransferase, ALT; alanine aminotransferase, ALP; alkaline phosphatase, GGT; γ-glutamyl transpeptidase, LDH; lactate dehydrogenase, UREAN; blood urea nitrogen, CREAN; creatinine, EGFR; estimated glomerular filtration rate, CRP; C-reactive protein, INR; international normalized ratio, APTT; activated partial thromboplastin time, FDP; fibrinogen degradation products, DDIMER; D-dimer, TP; total protein, ALB; albumin, PLR; platelet-to-lymphocyte ratio, NLR; neutrophil-to-lymphocyte ratio, TMP; temperature, NEUTLE; neutrophils, EOSLE; eosinophils, MONOLE; monocytes, LYMLE; lymphocytes.”

4. **I recommend assessing the discriminatory power of laboratory variables in predicting disease progression and mortality in COVID-19 patients by calculating the area under the receiver operating characteristic (ROC) curves (AUC). Using composite models could significantly improve the predictive accuracy (AUC values) for disease aggravation, thereby adding value to the manuscript**.

→ Thank you for pointing this out. We assessed the discriminatory power of Model I and Model II by generating receiver operating characteristic (ROC) curves and calculating the area under the curve (AUC) for each model. This explanation has been added to the Methods and Results section.

Revised text (Lines 36–38, Lines 45-46):

**Abstract**

“The predictive performance of two multivariate models—Model I (based on measurable clinical findings) and Model II (based on interview data)—was assessed using the area under the receiver operating characteristic curve (AUC).”

“The AUCs were 0.8928 for Model I and 0.8862 for Model II, indicating strong discriminatory power.”

Revised text (Lines 153–155):

**Statistical Analysis**

“The area under the receiver operating characteristic (ROC) curve (AUC) was calculated to evaluate the discriminatory power of each model.”

Revised text (Lines 252–254, Lines266-269):

**Results**

“Receiver operating characteristic (ROC) curves for both models are presented in Figure 3 (Panels A and B). The AUC was 0.8928 for Model I and 0.8862 for Model II, indicating good predictive performance for both models.”

“Figure 3.　The area under the receiver operating characteristic (ROC) curves (AUC) for multivariate analysis.

Figure 3-A. Model I

Figure 3-B. Model II”

5. **Although the authors discuss the inflammatory response, it would be helpful to further elaborate on why CRP, D-dimer, and procalcitonin were not statistically significant factors in this study.**

→ Thank you for this important comment. In our study, we excluded cases that were already severe at the time of admission as well as those that became severe on the same day. This allowed us to focus on patients who were non-severe upon admission and to identify risk factors associated with clinical deterioration despite standard medical care. The lack of statistical significance for CRP, D-dimer, and procalcitonin may be due to the fact that these markers are more reflective of an existing state, rather than predictive of future clinical worsening.

Revised text (Lines 303-307):

**Discussion**

“Procalcitonin and D-dimer levels were not significantly associated with aggravation in the univariate analysis. CRP, although significant in univariate testing, was not retained as a predictor in the multivariate model. This suggests that CRP, procalcitonin, and D-dimer—while elevated in severe cases (6, 19, 25, 26)—may not predict worsening at admission, as their elevations often reflect disease severity that has already progressed.”

6. **The Conclusion section requires improvement to better highlight the major findings of the study in relation to similar research. Additionally, future research directions should be briefly but clearly outlined. By addressing these concerns, the manuscript could be significantly strengthened in terms of scientific rigor and impact.**

→ Thank you for your thoughtful feedback. Based on the results of this study, we have added the following points to consider in the future research and what might be expected during future pandemics caused by emerging infectious diseases.

Revised text (Lines 48-51):

**Abstract**

“This study identified key risk factors for COVID-19 progression despite inpatient medical care among Japanese patients. These findings underscore the importance of early risk stratification for patients at high risk of deterioration despite treatment and may inform preparedness strategies for future respiratory pandemics.”

Revised text (Lines 336-345):

**Conclusion**

“This study investigated the factors associated with clinical deterioration in Japanese patients hospitalized with COVID-19 during the Wuhan strain phase of the pandemic, despite receiving appropriate medical care. The identified aggravating factors included early admission during the initial pandemic wave, older age, elevated BMI, high body temperature, anemia, dehydration and elevated levels of γ-GTP and LDH. These findings offer valuable insight into patient groups at higher risk of severe illness despite treatment, particularly in the context of future emerging respiratory viral infections. This information may contribute to pandemic preparedness and clinical risk stratification strategies. Further research is needed to assess whether these risk factors also apply to other emerging infectious diseases, such as novel strains of influenza.”

**Reviewer #2: The manuscript examined the aggravating factors for severe COVID-19 development despite medical care after admission among Japanese people during the COVID-19 pandemic. The topic is interesting, but the following points should be considered by the authors.**

→Thank you for your valuable comments. We have carefully addressed each of your points below and revised the manuscript accordingly to clarify and strengthen our analysis.

1. **The three sets or groups of analysis population FAS, FAS1, and FAS2 are really confusing; the authors need to make this point clear.**

→ Thank you for pointing this out. We agree that referring to FAS, FAS1, and FAS2 could be confusing. In the revised manuscript, we have simplified the terminology and focused our analysis on the FAS2 dataset. Specifically, we now refer to the analytical cohort as the *modified full analysis set* (*mFAS*) instead of FAS2. This change is reflected in the revised figure and throughout the text. The original dataset was designated the full analysis set (FAS), and after excluding patients who met the exclusion criteria or who were already in severe condition at the time of admission (or became severe on the day of admission), we defined the resulting cohort as *mFAS*, which was used for all statistical analyses.

Revised text (Lines 134-142):

**Statistical Analysis**

“The full analysis set (FAS) included all registered patients after applying exclusion criteria. For statistical analysis, a modified full analysis set (mFAS) was created by excluding patients who exhibited disease aggravation on the day of admission or before admission. Background characteristics and risk factors for worsening after admission were analyzed using the mFAS, as the focus was on predicting outcomes among patients who were not initially severe but who worsened despite medical care.

Univariate logistic regression models were applied to the mFAS to estimate odds ratios (ORs) and 95% confidence intervals (CIs) for each explanatory variable regarding the presence or absence of disease aggravation.”

Revised text (Lines 147-150):

“Two multivariate logistic regression models were developed using mFAS to assess the association of variables with aggravation risk. Model I estimated risk based on objective measures such as laboratory data and other measurable variables available at admission without requiring patient interviews.”

2. **Line 180-182 (A total of 2,884 patients were enrolled at 36 institutions throughout Japan that joined the JAID181 COVID registry. After excluding 25 ineligible patients and 45 cases of protocol deviation, 2,814 patients were analyzed), then the authors jumped to discussing about FAS, FAS1, and FAS2. I do not know which one is the study sample? If you have three study samples, then you could compare the parameters in your study samples.**

→ Thank you for highlighting this point. We acknowledge that the transition between data descriptions may have been unclear. As noted above, we have revised the figure and the Methods section to clearly define the progression from FAS to mFAS. The FAS included all eligible patients (n = 2,814), while the mFAS was derived by excluding patients who were already in a severe condition or worsened on the day of admission. This dataset (mFAS) serves as the primary sample for our analysis.

Revised text (Lines 171-178):

**Results**

“A total of 2,884 patients were enrolled from 36 institutions across Japan participating in the JAID-COVID registry. After excluding 25 ineligible patients and 45 with protocol deviations, 2,814 cases were included in the full analysis set (FAS). To identify factors associated with clinical aggravation despite medical care, patients who were already severe before admission (n = 45) and those who were severe at the time of admission (n = 211) were excluded. As a result, 2,598 patients were included in the modified full analysis set (mFAS), which served as the primary analytical cohort for this study (Figure 1). ”

Revised text (Lines 198-203):

“**Figure 1. Flow diagram of the study cohort.**

Registered cases: n = 2,884

Excluded cases: ineligible (n = 25), protocol deviation (n = 45)

Full analysis set (FAS): n = 2,814

Modified full analysis set (mFAS): n = 2,598 (excluding cases already severe before or at admission)”

3. **The authors must compare aggravating factors such as age, sex, body temperature, and laboratory data between patients who were discharged from the hospital and those who died from the disease. In this case, you will be able to prove which factor significantly contributed as an aggravating factor.**

→ Thank you for this important suggestion. In our study design, we classified patients who experienced clinical worsening—defined as requiring tracheal intubation, mechanical ventilation, ECMO, blood purification therapy, ICU admission, or who died—as the *aggravation* *group*. This group was compared with patients who were discharged without experiencing such deterioration. This approach allowed us to analyze risk factors associated with progression to severe disease despite receiving appropriate treatment. Although this definition was described in the “Patients and Study Design” subsection of the Methods section, it was not clearly stated in the Patient Data subsection. To improve clarity, we have now explicitly added the definition of “aggravation” to the Statistical Analysis section as well.

Revised text (Lines 115-119):

**Patient Data**

“Outcomes examined included aggravation (defined as requiring tracheal intubation, mechanical ventilation, extracorporeal membrane oxygenation [ECMO], initiation of blood purification therapy, intensive care unit [ICU] admission, or death), timing of hospital admission, COVID-19-associated complications, and disease duration (from symptom onset to recovery). ”

4. **The authors have to explain which medical care the patients had received.**

→ Thank you for your comment. We agree that it is essential to provide an overview of the medical care administered to the patients. Accordingly, we have added a description of the treatment protocols and types of care patients received during hospitalization as follows.

Revised text (Lines 189-196):

**Patient Data**

“Treatments administered during hospitalization included corticosteroids in 1,058 patients (40.7%), comprising methylprednisolone (n = 136), prednisolone (n = 65), hydrocortisone (n = 8), dexamethasone (n = 730), betamethasone (n = 2), and ciclesonide (n = 206). Antiviral drugs were given to 1,066 patients (41.0%), including remdesivir (n = 537) and favipiravir (n = 594). Additional interventions included oxygen therapy (n = 957; 36.8%), mechanical ventilation (n = 111; 4.3%), ECMO (n = 5; 0.19%), blood purification therapy (n = 8; 0.31%), catecholamines (n = 50; 1.9%), and anticoagulant therapy (n = 545; 21.0%).”

**I think the authors have to re-perform the statistical analysis appropriately and rigorously so that the tables are presented in an intelligible fashion and make sense.**

→ Thank you for this important observation. We have revised the tables for improved clarity and readability. In addition, as mentioned above, we have re-evaluated the statistical analysis and included new analyses.
